# Characterization of low pathogenicity avian influenza viruses isolated from wild birds in Mongolia 2005 through 2007

**DOI:** 10.1186/1743-422X-6-190

**Published:** 2009-11-05

**Authors:** Erica Spackman, David E Swayne, Martin Gilbert, Damien O Joly, William B Karesh, David L Suarez, Ruuragchaa Sodnomdarjaa, Purevtseren Dulam, Carol Cardona

**Affiliations:** 1 U.S. Department of Agriculture, Agricultural Research Service, Southeast Poultry Research Laboratory, Athens, GA 30605, USA; 2 Wildlife Conservation Society, 2300 Southern Boulevard, Bronx, NY 10460, USA; 3 Wildlife Conservation Society, 1008 Beverly Drive, Nanaimo, BC V9S 2S4, Canada; 4 State Central Veterinary Laboratory, Ministry of Food, Agriculture and Light Industry, Ulaanbaatar 210153, Mongolia; 5 University of California, Department of Population Health and Reproduction, Rm. 1383 Surge III, Davis, CA 95616, USA

## Abstract

**Background:**

Since the emergence of H5N1 high pathogenicity (HP) avian influenza virus (AIV) in Asia, numerous efforts worldwide have focused on elucidating the relative roles of wild birds and domestic poultry movement in virus dissemination. In accordance with this a surveillance program for AIV in wild birds was conducted in Mongolia from 2005-2007. An important feature of Mongolia is that there is little domestic poultry production in the country, therefore AIV detection in wild birds would not likely be from spill-over from domestic poultry.

**Results:**

During 2005-2007 2,139 specimens representing 4,077 individual birds of 45 species were tested for AIV by real time RT-PCR (rRT-PCR) and/or virus isolation. Bird age and health status were recorded. Ninety rRT-PCR AIV positive samples representing 89 individual birds of 19 species including 9 low pathogenicity (LP) AIVs were isolated from 6 species. A Bar-headed goose (*Anser indicus*), a Whooper swan (*Cygnus cygnus*) and 2 Ruddy shelducks (*Tadorna ferruginea*) were positive for H12N3 LP AIV. H16N3 and H13N6 viruses were isolated from Black-headed gulls (*Larus ridibundus*). A Red-crested pochard (*Rhodonessa rufina*) and 2 Mongolian gulls (*Larus vagae mongolicus*) were positive for H3N6 and H16N6 LP AIV, respectively. Full genomes of each virus isolate were sequenced and analyzed phylogenetically and were most closely related to recent European and Asian wild bird lineage AIVs and individual genes loosely grouped by year. Reassortment occurred within and among different years and subtypes.

**Conclusion:**

Detection and/or isolation of AIV infection in numerous wild bird species, including 2 which have not been previously described as hosts, reinforces the wide host range of AIV within avian species. Reassortment complexity within the genomes indicate the introduction of new AIV strains into wild bird populations annually, however there is enough over-lap of infection for reassortment to occur. Further work is needed to clarify how AIV is maintained in these wild bird reservoirs.

## Background

Surveillance of wild birds for avian influenza virus (AIV) has increased substantially worldwide in recent years due to the spread of the H5N1 high pathogenicity (HP) AIV among domestic and wild birds in Asia, Europe and Africa. Mongolia is an important location for H5N1 surveillance efforts as it supports large populations of wild birds from two major migratory flyways; the "East Asia-Australasian Flyway", and the "Central Asian Flyway" [1]. In addition, Mongolia has very little industrial poultry production. During 2005-2007 there were an estimated 100,000 chickens throughout the country and most birds are reared for egg production in moderately biosecure facilities located in urban centers [2,3]. No estimates for duck or turkey production have been established. This relative paucity of poultry production suggests that the presence of HPAIV would be the result of wild bird movement alone.

In order to better understand the ecology of AIV in wild birds, data from wild bird surveillance studies can be used to attempt to identify factors that correlate with AIV detection and isolation from wild birds, such as: reservoir species, bird health status, age, season and location. Additionally, phylogenetic data can help to elucidate how the virus is disseminated geographically. Here we report the detection, isolation and genetic characterization of nine LPAIVs isolated from wild birds in Mongolia from specimens collected in 2005 through 2007.

## Results

### Virus detection and isolation

A total of 2,139 swabs representing 4,077 individuals from 45 species were collected and tested (Table 1). Of these 443 samples representing 888 birds were tested by VI alone (samples collected in 2005) from which 4 low pathogenicity (LP) AIVs were isolated (0.9%) and one type-4 avian paramyxovirus (Table 2). All 4 were the H12N3 subtype (Table 2). All LPAIVs were isolated from fecal swabs. From this same set of samples one H5N1 HPAIV was isolated in 2005 from a dead Whooper swan (*Cygnus cygnus*) and will be characterized in depth in a separate report.

**Table 1:** Number of samples and birds tested by real-time RT-PCR and virus isolation.

			rRT-PCR		Virus Isolation		
				
Year	Total samples tested	Total no. of birds represented	No. pos/total tested	% pos	No. pos/total tested	% of total pos	% of rRT-PCR pos for VI
2005	443	888	NA^a^	NA	4/443	0.9	NA
2006	678	2,681	41/678	6.0	3/41	0.4	7.3
2007	1,018	508	49/1,018	4.8	2/49	0.2	4.1

Total	2,139	4,077	90/2,139	4.2	9/533	1.7	5.5^b^

a. NA = not applicable

b. Only includes data from 2006 and 2007, since samples collected in 2005 were not tested by rRT-PCR

**Table 2:** Low Pathogenic avian influenza virus isolates collected 2005-2007 from wild birds in Mongolia.

Isolate Name	Subtype	Species name	Bird health status	Bird age
BarHeadedGoose/Mongolia/143/2005	H12N3	*Anser indicus*	Unknown	Unknown
RuddyShelduck/Mongolia/P52/2005	H12N3	*Tadorna ferruginea*	Unknown	Unknown
RuddyShelduck/Mongolia/37/2005	H12N3	*Tadorna ferruginea*	Unknown	Unknown
WhooperSwan/Mongolia/232/2005	H12N3	*Cygnus cygnus*	Unknown	Unknown
BlackHeadedGull/Mongolia/1756/2006	H16N3	*Larus ridibundus*	Unknown	Unknown
BlackHeadedGull/Mongolia/1766/2006	H13N6	*Larus ridibundus*	Unknown	Unknown
RedCrestedPochard/Mongilia/1915/2006	H3N6	*Rhodonessa rufina*	Unknown	Unknown
MongolianGull/Mongolia/401/2007	H13N6	*Larus vagae mongolicus*	Dead	Juvenile
MongolianGull/Mongolia/405/2007	H13N6	*Larus vagae mongolicus*	Dead	Juvenile

Six hundred and seventy eight samples collected in 2006 were screened for AIV by rRT-PCR, of which 41 (6.0%) were positive. Viable AIV was isolated from 3 (7.3%) of the 41 rRT-PCR positive samples from 2006. One isolate was identified as the H3N6 subtype, one was H13N6 and one isolate was the H16N3 subtype (Table 2). A total of 1,018 samples collected in 2007 were screened by rRT-PCR for AIV, of which 49 (4.8%) were positive. Two H13N6 AIVs (4.1% of the 49 rRT-PCR positives) were isolated from the 2007 samples. Although virus isolation was not attempted on all samples from 2006 and 2007 because they were initially screened with rRT-PCR first, the rates of isolation from the total numbers of samples collected were 0.4% for 2006 and 0.2% for 2007 (assuming none of the rRT-PCR negative samples would have been virus isolation positive).

### Virus detection by species, age and bird health status

Overall 19 species were positive for AIV by rRT-PCR and viable virus could be isolated from 6 species (Table 2 and Additional file 1). Age and health status could only be assigned for birds that were sampled individually. The 90 samples that tested positive by rRT-PCR comprised 36 pooled fecal samples and swabs from 53 individual birds (one bird tested positive on both upper respiratory and cloacal swabs). In total 17 juvenile (9.8%, n = 173) and 36 adult (11.2%, n = 321) birds sampled individually tested positive by rRT-PCR. The difference in proportion of the number of positive adults and juveniles was not statistically significant (Fishers exact test). The health status of individual birds from which rRT-PCR positive swabs were collected included 45 healthy, 1 sick and 7 dead birds. All isolates obtained in 2005 and 2006 were derived from fecal samples therefore age and health status could not be determined, while both isolates in 2007 were collected from dead juveniles.

### Phylogenetic analysis

**Figure 1:**
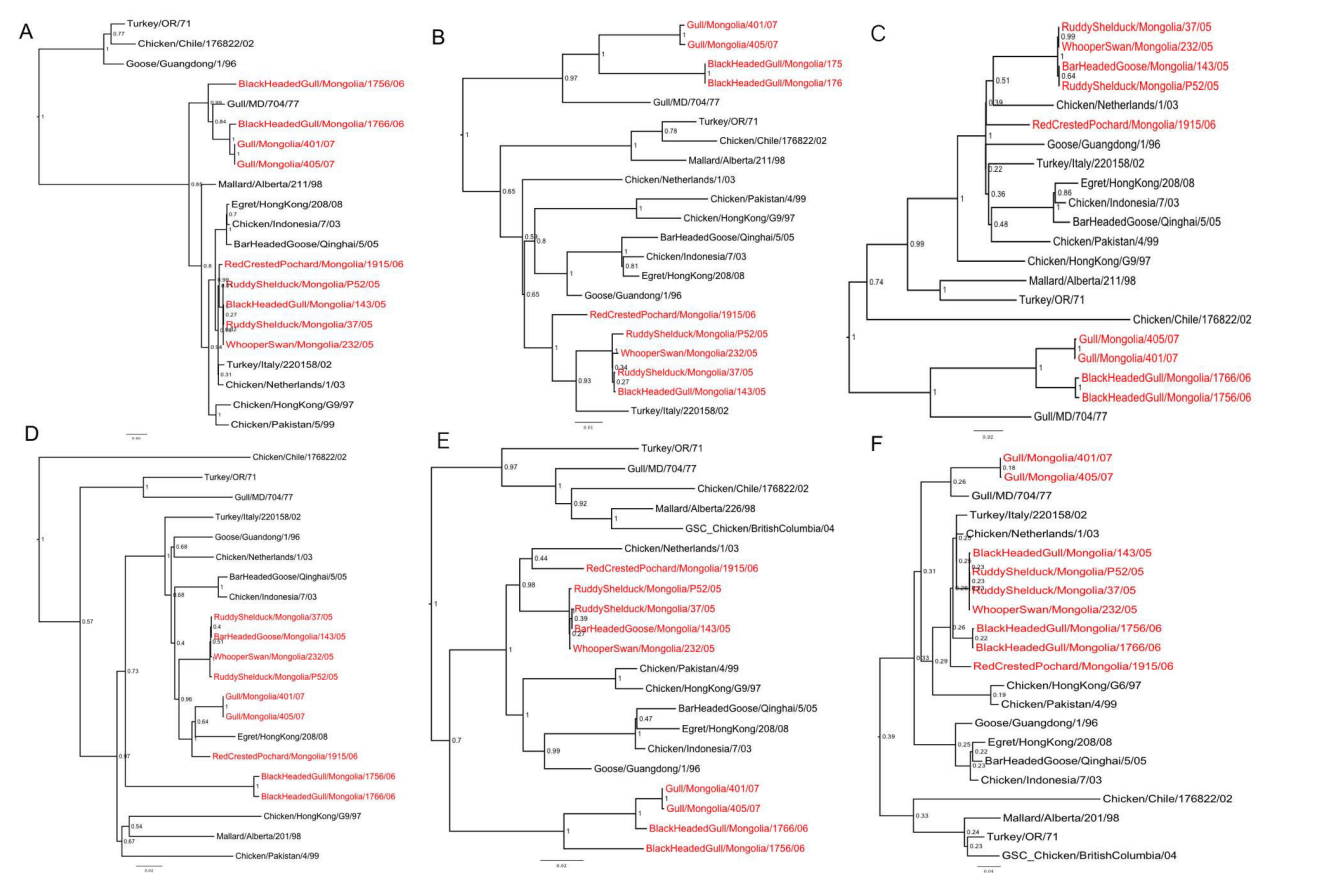
**Phylogenetic trees of internal protein genes**. Trees include all avian influenza virus isolates collected from wild birds in Mongolia 2005-2007 and selected reference isolates. Trees are shown for all 8 segments of each isolate as follows: A) NS, B) M, C) NP, D) PA, E) PB1 and F) PB2. Trees were constructed with merged duplicate runs of BEAST v. 1.4.8 using HKY substitution, empirical base frequency, Gamma heterogeneity, codon 2 partitions, relaxed lognormal clock, Yule Process tree prior with default operators with UPGMA starting tree and MCMC length of 10^7^. Posterior values are shown at the nodes.  Isolates collected during this study are shown in red font.

**Figure 2:**
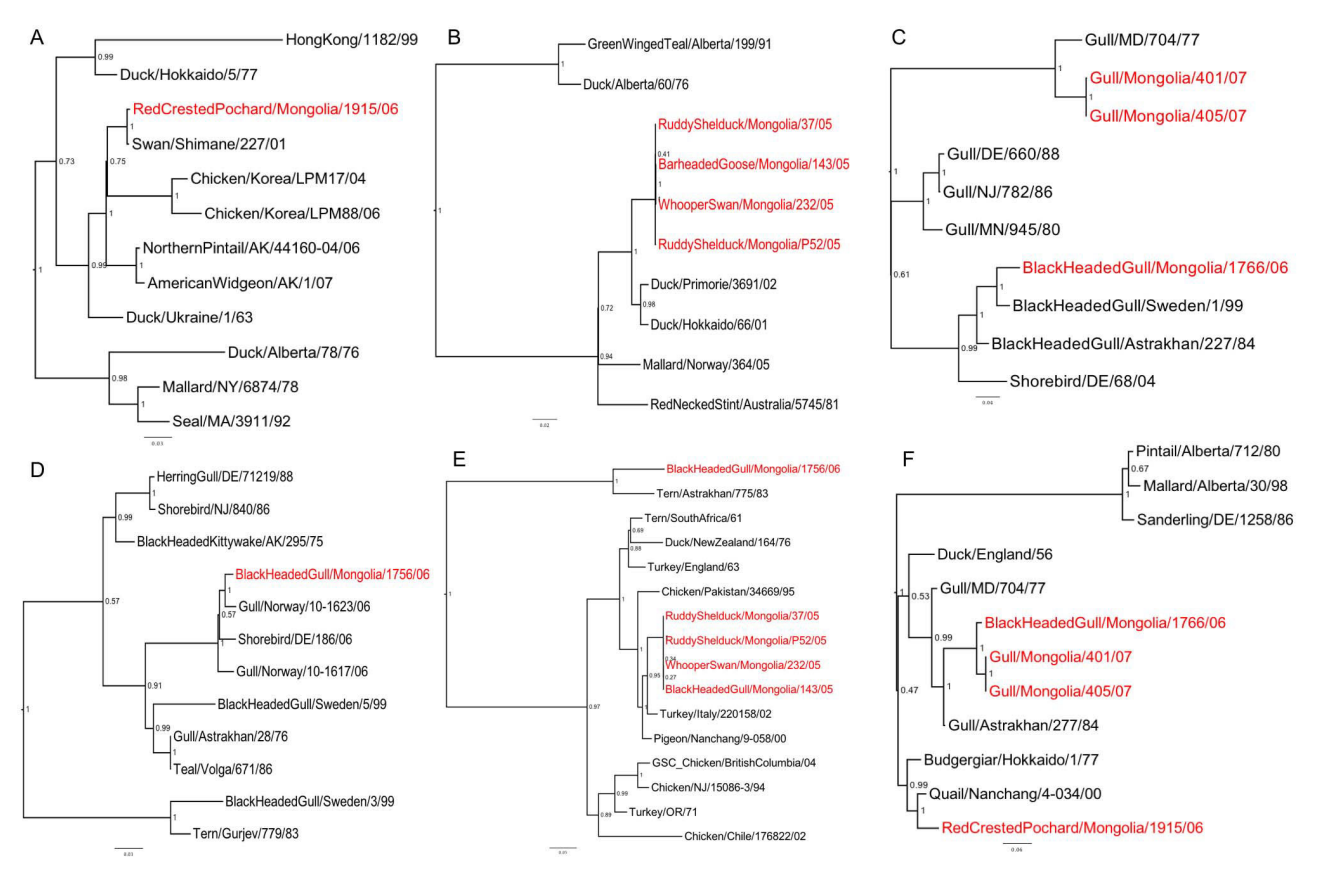
****Phylogenetic trees of HA and NA genes**. **Trees include all the HA and NA genes from avian influenza virus isolates collected from wild birds in Mongolia 2005-2007 with selected reference isolates. Trees are shown for all 8 segments of each isolate as follows: A) H3, B) H12, C) H13, D) H16, E) N3 and F). N6. Trees were constructed with merged duplicate runs of BEAST v. 1.4.8 using HKY substitution, empirical base frequency, Gamma heterogeneity, codon 2 partitions, relaxed lognormal clock, Yule Process tree prior with default operators with UPGMA starting tree and MCMC length of 10^7^. Posterior values are shown at the nodes. Isolates collected during this study are shown in red font.

The coding sequences of the full genomes of all isolates were sequenced and analyzed phylogenetically. All eight genes of all nine Mongolian viruses were most closely related to Asian or European wild-bird lineage viruses (Figures 1 and 2), although there was variation among the 8 gene segments.

Among the nine LPAIV viruses isolated during this study there was some genetic variation observed among all eight gene segments, however some of the viruses isolated in the same year which were also the same HA and NA subtype had very closely related genomes (Figures 1 and 2); for example all 8 gene segments from the 4 H12N3 isolates from 2005 were closely related to each other (98.0% or higher identity). Similarly all 8 gene segments of the 2 H13N6 viruses from 2007 were closely related to each other (99.0% or greater identity). Evidence of multiple lineages and reassortment was seen among the remaining isolates; the internal protein genes (NS, M, NP, PA, PB1, PB2) from the 2006 H13N6 isolate (BlackHeadGull/1766/06) were more closely related to the BlackHeadGull/1756/06 H16N3 isolate (minimum of 99.2% identity), than to the 2007 H13N6 viruses. The H3N6 virus isolated in 2006 (RedCrestedPochard/1915/06) was most closely related to the 2005 viruses in the M, NS, NP, PA and PB1 genes (> 97% identity between RedCrestedPochard/1915/06 and the 2005 viruses) and the other 2006 viruses in the PB2 with 93.6% identity.

## Discussion

Efforts to monitor wild birds have increased worldwide in recent years based on concern with the possibility that wild birds would disseminate the Asian H5N1 HPAIV. Mongolia provides a good location for wild bird monitoring because there is very little domestic poultry production. Therefore the detection of the Asian H5N1 HPAIV in resident or migratory birds would likely be due to wild birds and not spillover from infected poultry. Equally important, this study also provides an opportunity to collect additional data on the dissemination of LPAIV in wild birds in Asia by contributing data to help establish the genetic relationships among wild bird origin AIVs.

Overall trends in factors that correlate with AIV detection and isolation from wild birds, such as species, and bird health status correlate between this study and trends reported for other locations. Real-time RT-PCR positive specimens were collected from a total of 19 species, from which viable avian influenza virus was isolated from 6 species. Sampling was targeted to waterbirds of orders Anseriforme and Charadriiforme, but also included representatives of Gaviformes, Podicepiformes, Gruiformes, Falconiformes, Coraciformes and Passeriformes. With the possible exception of two Passeriformes (*Luscinia svecica *and *Calandrella cheleensis*) which were rRT-PCR positive and virus isolation negative, AIV has been isolated from or detected in samples from all the other species that were positive for virus isolation or detection in this study [4,5].

Since LPAIV, as defined by the world animal health organization [6], does not normally cause disease in wild bird species [7], it was expected that most of the birds from which the isolates were obtained would be healthy; however 2 virus isolates and 6 virus isolation negative, rRT-PCR positive samples were collected from dead birds. Without the support of pathological findings, it is not possible to determine if AIV contributed to the death of these birds, but it is unlikely and is probably coincidental. Since detection of the Asian H5N1 HPAIV, which can lead to mortality in some aquatic bird species in the wild [8,9], was a primary interest in this study, sample collection was biased to dead birds, if present.

One possible exception to trends reported in most previous studies are AIV isolation rates by age. The rates frequently reported as being higher in juvenile than adult wild birds [10-13], here an essentially equal proportion (statistically the same) of adult birds (11.2%, n = 321) were found to be positive by rRT-PCR as juveniles (9.8%, n = 173). The importance of this is unclear since they were only rRT-PCR positive. Also the relative proportions of adults to juveniles that were sampled in each species were biased, with adult samples biased to Anseriforme species (that might be expected to show higher prevalence of AIV), whereas *Phalacrocorax carbo*, were over-represented among juveniles (and might be expected to exhibit lower AIV prevalence rates). The age was recorded for too few birds that were positive for virus isolation to draw conclusions.

In general AIV isolation and detection rates in wild birds vary substantially by year, season, location and species [12-17]. The rates of isolation observed here, both overall and for individual years, was below 1%, however, since this study was only conducted for three years, there is not enough data to establish long-term trends for these species in Mongolia.

## Conclusion

The broad host range of AIV in avian species has been well described and is reinforced by this report which adds two species which have not been previously identified as hosts (although only by rRT-PCR). Further work would be needed to establish whether these species may serve as reservoirs.

The genetics of the virus can offer insight into the dissemination and mixing of virus populations in wild bird reservoirs and can offer insight into the evolution of AIV. The nine LPAIVs isolated from wild birds in Mongolia showed a great deal of genetic variation in all 8 gene segments and although there was some grouping of genes from viruses isolated in the same year, there was also evidence of reassortment and it appeared that lineages were not maintained from year to year indicating that the virus is being re-introduced with some over-lap of infection. Some of the genetic differences may also be attributed to species of origin, since the viruses do also group by species, or by species groups that share habitat. Therefore the broad differences in lineages from year to year may be due to what is being carried by a species and how much that species is able to transmit the virus to other species at a location. It is clear that numerous lineages of AIV move through Mongolia, attesting to the diversity of AIV populations in wild birds. Further work is needed to illuminate the details of AIV ecology in these species and habitats.

## Materials and methods

### Specimen collection

**Figure 3:**
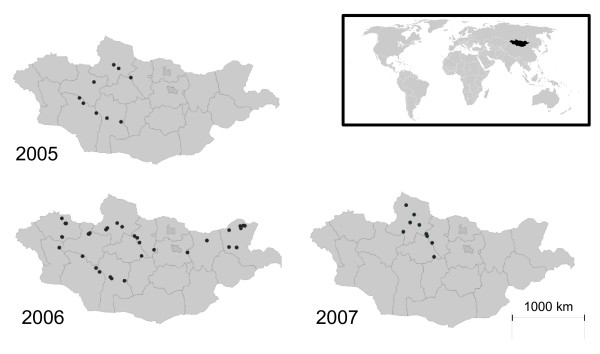
Location of the sampling sites in Mongolia by year

Samples were collected from a total of 43 locations across Mongolia (Figure 3) during three visits to the country in July-August 2005, July-October 2006 and April-October 2007. The primary reason for the variation in changes of sampling sites related to the work being part of the national surveillance response to HPAI H5N1, rather than a purely hypothesis driven study. In 2005 when we mounted our first sampling expedition to the country, our objective was to determine whether there was any evidence that the recent outbreak reported at Qinghai [8] might have spread elsewhere along the flyway. By the time we had returned in 2006, our objective was slightly different, as by that time the country had confirmed three outbreaks of HPAI and the question was more one of how widespread the virus had become (hence the wider geographical range). By 2007 there had been no further outbreaks reported in Mongolia, so we reduced our search area to the lakes within the general vicinity of, and including the national outbreak lakes. No commercial poultry was located near sampling sites.

Sampling strategies included the collection of fresh environmental fecal samples and individual swabs from sick or dead birds and from live healthy birds captured using techniques appropriate for each species. Fresh environmental fecal swabs were collected from single-species congregations of birds to assure correct identification, and placed individually, or pooled in groups of five in cryo-vials containing viral transport media consisting of 1-2 ml of brain heart infusion broth containing 5 μg/ml amphotericin B, 10,000 units/ml penicillin, and 1,000 μg/ml gentamycin sulphate. Cloacal swabs and swabs from the upper respiratory tract (collected from the oropharynx or trachea depending on bird size) were collected from individual live, sick and dead birds and placed individually in cryo-vials containing viral transport medium. The ages of individual birds were estimated based on plumage characteristics and their sex was determined using differential plumage, or extrusion of genitalia (ducks, geese and swans). Samples were maintained at 4°C upon collection then frozen in liquid nitrogen within 4 hours of collection. Care was taken to maintain the cold-chain for specimens throughout transport and once in the lab all samples were stored at -70°C or below until processed.

### Sample processing

Sample processing was modified from year to year to accommodate logistical changes and in an attempt to minimize false negative results due to virus degradation. Although these minor modification somewhat reduce the ability to compare data year-to-year, it was deemed that the increase in accuracy was more important. Samples collected in 2005 were collected in viral transport media and all specimens were tested only by virus isolation (VI); rRT-PCR was not attempted with samples from 2005. Samples collected in 2006 were preserved in viral transport media in 2006, tested by rRT-PCR for type A influenza as described below and VI was subsequently attempted with all rRT-PCR positive samples. Samples collected in 2007 were split into a vial of guanidine isothiocyanate and a duplicate vial of viral transport media. The samples in guanidine isothiocyanate were screened by rRT-PCR as described below; then VI was subsequently attempted with all rRT-PCR positive samples on the duplicate samples that had been preserved in viral transport media.

### Screening of swabs for AIV by rRT-PCR

Samples from 2006 and 2007 were processed at different laboratories, therefore the procedures were not identical. Samples collected in 2006 were processed at Southeast Poultry Research Laboratory, USDA-ARS as follows: RNA was extracted from swabs using a procedure optimized for oral and cloacal swab samples as previously described [18]. A previously reported rRT-PCR test that targets the type A influenza matrix (M) gene was run on the Smart Cycler (Cepheid, Inc., Sunnyvale, CA) real-time PCR instrument as previously described [19]. An internal positive control [20] was included to ensure that inhibitors were not causing false negative results; any samples that had both a negative M gene test and a negative internal control result were not counted as tested samples. Samples that were positive for the influenza M gene were processed for virus isolation.

Samples from 2007 were processed at the University of California, Davis as follows: RNA was extracted from swab samples in guanidine isothiocyanate using the MagMAX-96 Viral Isolation Kit (Ambion Inc. Austin, TX) in accordance with the manufacturers instructions. A rRT-PCR which targets the M gene was conducted as described by Runstadler, et al. [21] which was the standard test in this lab and run on an AB 7500 Real-Time PCR System (Applied Biosystems, Foster City, CA).

### Virus isolation and subtype identification

Virus isolation was attempted with swab material using 10-day old specific-pathogen-free (SPF) embryonating chicken eggs using established procedures [22]. Hemagglutinating agents from VI attempts were confirmed as type A influenza by commercial antigen capture assay (BinaxNOW Flu A and B, Inverness Medical, Inc. Portland, ME). The subtypes of influenza positive samples were determined by gene sequencing of the HA and NA genes as described below. If virus isolation attempts were unsuccessful, no further characterization was conducted on rRT-PCR positive, virus isolation negative samples, because of the difficulty of amplifying an unknown HA and NA subtypes with the low amount of viral RNA frequently obtained from swab material; it was considered to be too resource intensive for the amount of information that would be gained.

### Virus genome sequencing and analysis

Full genome sequencing of all isolates was performed as previously described [23]; Genbank accession numbers: GQ907286-GQ907357. Phylogenetic analysis was performed by aligning the nucleotide sequences of genes from the Mongolian viruses and selected reference isolates for the different lineages for each gene and aligning them with either ClustalV or ClustalW (Lasergene 7.1, DNASTAR, Madison, WI). Trees were constructed with merged duplicate runs of BEAST v. 1.4.8 [24] using HKY substitution, empirical base frequency, Gamma heterogeneity, codon 2 partitions, relaxed lognormal clock, Yule Process tree prior with default operators with UPGMA starting tree and MCMC length of 10^7^.

## Abbreviations

AIV: avian influenza virus, HA: hemagglutinin; HP: high pathogenicity; LP: low pathogenicity; NA: Neuraminidase; rRT-PCR: real-time reverse-transcription polymerase chain reaction; SPF: specific pathogen free; VI: virus isolation

## Competing interests

The authors declare that they have no competing interests.

## Authors' contributions

ES generated sequence data, conducted the rRT-PCR testing on the 2006 specimens and performed the phylogenetic analysis. DES participated coordinating the study and conducted the virus isolation. MG, DOJ and WBK designed the study, collected the specimens and ornithological data. RS and PD participated in collecting the specimens and ornithological data. DLS generated sequence data. CC conducted the rRT-pCR testing of samples collected in 2007. All authors have read and approved the final manuscript.

## Supplementary Material

Additional file 1 **Summary of rRT-PCR results for species testing positive for type A influenza**. Summary of rRT-PCR testing by species, bird age and health status. Click here for file
