# Supplementary material for: Characterization of low pathogenicity avian influenza viruses isolated from wild birds in Mongolia 2005 through 2007
Source: Virol J. 2009 Nov 5;6:190. doi: 10.1186/1743-422X-6-190 (PMC2781007; doi:10.1186/1743-422X-6-190)
Supplement: Additional file 1 — Summary of rRT-PCR results for species testing positive for type A influenza. Summary of rRT-PCR testing by species, bird age and health status. [file 1743-422X-6-190-S1.pdf]

Additional file 1. Summary of rRT-PCR results for species testing positive for type A influenza.

Species common name	Species scientific name	Fecal pools[1]	Total birds	Adults	Juveniles	Healthy	Sick	Dead
Whooper Swan	*Cygnus cygnus*	9 (2)[2]	129 (13)	129 (13)		129 (13)		
Mixes swan spp.	*Cygnus spp.*	21 (6)						
Swan Goose	*Anser cygnoides*	9 (0)	23 (1)	23 (1)		23 (1)		
Bean Goose	*Anser fabalis*		21 (4)	21 (4)		21 (4)		
Bar-headed Goose	*Anser indicus*	14 (1)	122 (14)	122 (14)		118 (14)	1 (0)	3 (0)
Ruddy Shelduck	*Tadorna ferruginea*	96 (3)	34 (4)	21 (2)	13 (2)	33 (4)		1 (0)
Common Shelduck	*Tadorna tadorna*	9 (1)						
Common Teal	*Anas crecca*	24 (1)						
Red-crested Pochard	*Rhodonessa rufina*	39 (3)						
Common Pochard	*Aythya ferina*	26 (2)						
Common Goldeneye	*Bucephala clangula*		1 (1)	1 (1)			1 (1)	
Demoiselle Crane	*Grus virgo*	3 (0)						
Little-ringed Plover	*Charadrius dubius*		2 (1)	1 (1)	1 (1)	2 (1)		
Northern Lapwing	*Vanellus vanellus*	20 (1)	1 (1)		1 (1)			1 (1)
Mongolian Gull	*Larus vagae mongolicus*	61 (5)	44 (5)	1 (1)	43 (5)	3 (0)		41 (5)
Black-headed Gull	*Larus ridibundus*	62 (7)						
White-winged Tern	*Chlidonias leucopterus*		4 (1)		4 (1)	4 (1)		

Species common name	Species scientific name	Fecal pools[1]	Total birds	Adults	Juveniles	Healthy	Sick	Dead
Great Cormorant	*Phalacrocorax carbo*	126 (4)	109 (6)	1 (1)	108 (6)	96 (6)	5 (0)	8 (0)
Bluethroat	*Luscinia svecica*		3 (1)		3 (1)	3 (1)		
Asian Short-toed Lark	*Calandrella cheleensis*		1 (1)	1 (1)				1 (1)
	Total	513 (36)	494 (53)	321 (36)	173 (17)	432 (45)	7 (1)	55 (7)
	Percentage positive	7.0%	10.7%	11.2%	9.8%	10.4%	14.3%	13.0%

1. Fecal pools contain 5 individual swabs.
2. Number of pool tested (number of type A influenza positive pools).
